# A systematic review of models to predict recruitment to multicentre clinical trials

**DOI:** 10.1186/1471-2288-10-63

**Published:** 2010-07-06

**Authors:** Katharine D Barnard, Louise Dent, Andrew Cook

**Affiliations:** 1 National Institute for Health Research Evaluation, Trials and Studies Coordinating Centre (NETSCC), University of Southampton, UK

## Abstract

**Background:**

Less than one third of publicly funded trials managed to recruit according to their original plan often resulting in request for additional funding and/or time extensions. The aim was to identify models which might be useful to a major public funder of randomised controlled trials when estimating likely time requirements for recruiting trial participants. The requirements of a useful model were identified as usability, based on experience, able to reflect time trends, accounting for centre recruitment and contribution to a commissioning decision.

**Methods:**

A systematic review of English language articles using MEDLINE and EMBASE. Search terms included: randomised controlled trial, patient, accrual, predict, enrol, models, statistical; Bayes Theorem; Decision Theory; Monte Carlo Method and Poisson. Only studies discussing prediction of recruitment to trials using a modelling approach were included. Information was extracted from articles by one author, and checked by a second, using a pre-defined form.

**Results:**

Out of 326 identified abstracts, only 8 met all the inclusion criteria. Of these 8 studies examined, there are five major classes of model discussed: the unconditional model, the conditional model, the Poisson model, Bayesian models and Monte Carlo simulation of Markov models. None of these meet all the pre-identified needs of the funder.

**Conclusions:**

To meet the needs of a number of research programmes, a new model is required as a matter of importance. Any model chosen should be validated against both retrospective and prospective data, to ensure the predictions it gives are superior to those currently used.

## Background

Large scale multicentre randomised controlled trials (RCTs) are regarded as the gold standard in rigorous, robust clinical research. Participants are recruited across a number of centres and are randomly assigned to control or intervention groups. Rates of participant recruitment, however, are not as straightforward as it may seem and this can have an enormous impact on the planning, execution and funding of trials. Failure to recruit sufficient numbers of participants, or extended delays in recruitment can have serious implications for the success or otherwise of the trial.

Research funders usually require applicants to calculate how many participants they require to answer their identified research questions, and how long they will need to recruit those participants into their study. In deciding how long they will require to accrue participants, trialists are often over optimistic - in 2007 Campbell found that less than one third of publically funded trials managed to recruit according to their original plan [1]. In some sectors this problem affects up to 80% of studies [2]. Careful planning and preparation can give better estimates of how realistic a particular accrual target is, and how long it will take to recruit the required number of patients.

As the leading public funder of clinical trials in the United Kingdom, the NIHR Health Technology Assessment (HTA) programme has an interest in optimising its investment in trials - and one way in which it can do this is by ensuring good estimates of timescales for research are available at the time of taking funding decisions.

In order to get good estimates, accurate and realistic patient recruitment predictions are required. Unfortunately, most HTA applicants adopt the unconditional approach [3] to recruitment prediction - suggesting that all their planned centres will start recruiting to their maximum capacity on day one. These centres tend not to be a pre-existing trial network, but are recruited on an ad-hoc basis for the needs of the trial. Given the difficulties with ethical and research governance approval at individual centres, this rarely happens.

As the application process is competitive - multiple teams compete to be commissioned to answer a single question - it might be expected that applicants overestimate their ability to recruit patients to trials, in order to make their applications appear more attractive. This appears to be borne out by 70% of trials failing to meet their original recruitment targets and requiring either additional time and money, or reconsidering the power of their study [4].

In order to facilitate the work of the HTA programme, and other research funders, the authors set out to investigate whether it would be feasible to provide a model for applicants intending to carry out pragmatic clinical trials to use to improve the prediction of recruitment to trials. The project to deliver this tool was divided into two phases. The first phase was a systematic literature review to identify currently existing tools and assess whether they met the programmes requirements. The objective of the second phase was to produce and test a predictive model for the HTA programme - through either building on a model found in phase 1, or starting from scratch.

This paper reports phase one - the systematic literature review.

## Methods

For a predictive recruitment model to be useful to a wide group of stakeholders - including researchers and funders - it must exhibit certain characteristics, i.e. be:

1. *Simple to Use and Understand - *Applicants should be able to describe their proposed trial, and the model should provide the likely recruitment profile.

2. *Can adapt to epidemiological changes *- reflecting the research community's previous experience of the ability of studies to recruit patients and centres in the area of the trial.

3. *Can adapt to environmental changes *- changes affecting the conduct of trials in general for example, changes in ethical approval processes or regulatory requirements which then alter the lead time required to add a centre to a trial.

4. *Able to take account of centre recruitment - *The promptness of centres starting to recruit patients is a significant driver for overall recruitment in multicentre trials.

5. *Able to inform commissioning decisions *- The model should provide an expected recruitment period to allow proposals to be compared, rather than a probabilistic analysis.

We set out to identify existing models, and assess them against these criteria.

To identify currently existing models, a systematic literature review was conducted. Medline and Embase were searched in July 2008. The following truncated free text terms were searched in the title and abstract fields for: ((subject$ or patient$ or projected or accrual or fail$ or stop$ or clos$ or shut or predict$ or estimat$) adj5 (recruit$ or particip$ or enlist$ or enrol$)); plus MeSH terms "Patient Selection" or "Sample Size". Model types and statistical methods were also searched for: models, theoretical; models, organizational; models, statistical; Bayes Theorem; Decision Theory; Monte Carlo Method; Stochastic Processes; Clinical Trials Data Monitoring Committees; Data Interpretation, Statistical; Statistics; Poisson Distribution; normal distribution.

The papers identified by the search were reviewed by KB and AC to ensure all appropriate identified studies were included. Non-English language papers were excluded, due to a lack of resource for translation. Papers were excluded if they did not discuss using modelling techniques to predict patient recruitment to individually randomised clinical trials.

**Figure 1:**
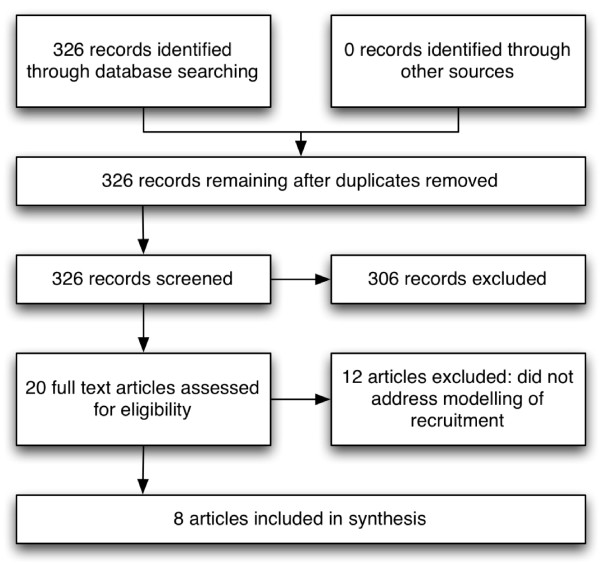


In order to identify candidate papers in the grey literature which may not be included in standard databases subsidiary searching was carried out using the Google search engine. No papers were found which were not identified through the formal search strategy (see Figure 1).

Data was extracted from the identified papers using a pre-defined data-extraction form. Data was extracted by KB initially and was then checked by LD and AC.

The categories used in the form were

1. question the identified model was designed to address, where not explicitly stated in the paper this is as determined by the reviewers

2. applicability of the model to the needs of the HTA programme and other pragmatic trial funders (eg to multi-centre studies, ad-hoc centres)

3. Approach of the model - the mathematics of the model, what data was used to populate the model, how it was validated

4. Centre Recruitment - did the model consider recruitment of both centres and individual patients?

## Results

Three hundred and twenty six papers were identified in the initial search, none of these were duplicates.

Following the initial screen of abstracts, 306 were excluded as the title indicated they did not address modelling of trial recruitment. Full papers were assessed for 20 studies.

Following the full paper review, 12 studies were excluded as they did not address modelling of trial recruitment.

A summary of this process is shown in the PRISMA diagram in Figure 1[5]

The 8 studies included are summarised in the summary of study data extraction (Additional file 1).

Of the 8 studies examined, there are five major classes of model discussed: the unconditional model, the conditional model, the Poisson model, Bayesian models and Monte Carlo simulation of Markov models (see Table 1 for a summary of these types of model).

**Table 1:** Definitions of the different types of models

Model	Definition
Unconditional model	The unconditional approach estimates the accrual period by dividing the pre-specified sample size by the number of patients they expect to recruit across all centres each month (e.g. assume a trial requires 400 participants, its estimated centres will recruit 10 participants per month. According to the unconditional model this implies it will take 40 months to recruit all 400 participants. ^2^

Conditional model	The conditional model allows the expected recruitment in any given month to vary, depending on other conditions in the trial such as how many centres are available to recruit (e.g. assume a trial requires 400 participants and that its expected centres 1 and 2 will start recruiting in month 1 and will recruit 5 participants per month each. Also assume its expected that centre 3 will start recruitment in month 6 and will recruit 10 participants per month. According to the conditional model this implies it will take 22.5 months to recruit all participants).^2^

Possion model	The poisson models, assumes the rate that participants are recruited varies according to a poisson distribution. The number of participants recruited within a given month is simulated using a random number generator, from the poisson distribution with mean λ, where λ is the mean number of participants that trialists specify they expect to recruit each day/month. The **Poisson distribution **is a discrete probability distribution that expresses the probability of a number of events occurring in a fixed period of time if these events occur with a known average rate (λ) and independently of the time since the last event. P(X_t _= n) = (e^-λt ^(λt)^n ^)/n!^2^

Bayesian model	A Bayesian analysis starts with a "prior" probability distribution for the value of interest (for example, the recruitment of participants into a trial)--based on previous knowledge--and adds the new evidence as data accumulates (via a model) to produce a "posterior" probability distribution. ^10^

*Monte carlo simulation Markov model*	A type of quantitative modeling that involves a specified set of mutually exclusive and exhaustive states (e.g., of a given health status), and for which there are transition probabilities of moving from one state to another (including remaining in the same state). In this case a participant moving from a contacted state to a recruited state in a specified time period. Typically, states have a uniform time period, and transition probabilities remain constant over time.
	
	Values are randomly generated from a uniform distribution, if the value generated is less than equal to the transition probability assumed a participant is said to be recruited in that time period.
	Monte Carlo simulation considers random sampling of probability distribution functions as model inputs to produce hundreds or thousands of possible outcomes instead of a few discrete scenarios. The results provide probabilities of different outcomes occurring.
	
	Markov chain monte carlo simulations use, monte carlo simulation (random number generation) to decide on the transition probability and whether a participant moves from one state to another (is recruited in this time period or not).^8,11^

Carter's 2005 paper [3] considers the conditional model, a deterministic approach is also discussed by Moussa [6]. The conditional model, is a development of the unconditional model which is commonly used by applicants for NIHR HTA programme grants. The conditional model allows the expected recruitment in any given month to vary, depending on other conditions within the trial such as how many centres are available to recruit. This more closely matches the real life experience of multi-centre trials relying on ad-hoc centre recruitment. Using relatively simple calculations this model can easily be constructed within a spreadsheet.

Three papers considered Poisson modelling [3,7,8]. Of these, two are linked, with one addressing the theory [4] and the other discussing a practical use of the method [4]. Carter (2005) [3] presents the effect of these models on a trial protocol in development. As the prediction models become more sophisticated, the predicted accrual periods become longer. It should be noted that the mean predicted accrual periods for the conditional and Poisson approaches are very similar at 23 months and 23.2 month respectively. Anisimov [7] takes a similar approach to Carter (2005), in that he constructs a Poisson based model, but allows the recruitment rate to vary according to a Gamma distribution. He makes no allowance for varying the number of recruiting centres over time.

Williford considers the relative advantages of the Poisson and Bayesian approaches in monitoring ongoing recruitment within a trial and predicting future goals [8].

Gajewski develops the Bayesian approach of Williford to include establishing prior probabilities without requiring accrual data from a specific trial [9]. His initial prior probability is derived from either previous experience in similar trials, or from clinical opinion based on epidemiology. As more trial specific accrual data becomes available, the prediction can be refined by bringing this data into the calculation.

Abbas [10] uses a number of Markov models to explore the maximisation of recruitment of patients in a minimum amount of time. He starts from the assumption of a fixed set of centres. He does present the only approach that deals with depleting willing trial participants from a population and the effect this has on the length of an accrual period. Abbas does not however consider the recruitment of trial centres - which could have an effect on reducing the effect of exhaustion of the trial pool.

Hadich [11]uses a time series approach to analyse an existing set of data retrospectively, to determine if it would have been possible to predict recruitment to a trial. This time series model is not validated prospectively.

The characteristics of the identified models were compared to the criteria initially established, a summary of the comparison is shown in Table 2.

**Table 2:** Match between identified models with requirements of HTA programme

Paper	Model Type	Simplicity	Can adapt to epidemiological changes	Can adapt to environmental changes	Centre Recruitment	Could inform commissioning Decisions
Carter (2004)	Simulation using Poisson distribution	Y	P	Y	Y	Y

Carter (2005)	Unconditional	Y	Y	N	N	Y
		
	Conditional	Y	Y	Y	Y	Y
		
	Simulation using Poisson distribution	Y	P	Y	Y	Y
		
	Simulation using Poisson distribution with average recruitment rates (λ) varied according to a uniform distribution	Y	P	Y	Y	Y

Anisimov (2007)	Poisson process with recruitment rates (λ) viewed as a sample from a gamma distribution	N	Y	Y	N	P

Moussa (1984)	Conditional	Y	Y	Y	N	Y

Williford (1987)	Poisson	N	Y	Y	N	N
		
	Negative binomial (Poisson process with recruitment rates (λ) viewed as a sample from a gamma distribution)	N	Y	Y	Y	N
	
	Lees contagious poisson	N	Y	Y	Y	N

	Bayesian - prior distribution is possion-gamma, posterior is gamma	N	Y	Y	N	N

Gajewski (2007)	Bayesian - prior distribution is the inverse gamma, likelihood is the exponential distribution, posterior distribution is the inverse gamma	N	Y	Y	P	Y

Abbas (2007)	Markov	N	P	Y	N	Y

Hadich (2001)	Time series	N	P	N	N	N

Y = Criterion met

N = Criterion not met

P = Posssibly: Criteron could be met, dependent on circumstance

Comparing the different model types identified in this literature it becomes clear that the unconditional model does not fulfil sufficient of the pre-identified criteria because it is too simple and cannot take account of changes in centre recruitment or regulatory environment. The conditional model has promise because it satisfies all the criteria, however it has no fixed structure so a new model must be constructed for each use. The poisson model is promising, as using Carter 2004's illustrations you can allow lambda to vary for different periods of time in the trial as additional centres start recruiting. This involves simulating and combining data from a number of different poisson distributions which complicates the model slightly. The possion model can be further enhanced by allowing lambda to vary according to a gamma or uniform distribution as illustrated by Carter (2005), Amnisov and Williford. It is not clear from the literature exactly how centre recruitment can be taken into account in the poisson-gamma model and if this additional level of complexity is necessary. Baysian models rely on data being available to establish prior probabilities of recruitment which can be updated over the course of the study, making them more challenging to use for commissioning decisions, as they become more accurate as more trial data becomes available. Therefore, they are of more benefit when taking continuation decisions over the course of a trial. Markov models require structural model changes each time they are used, they are comparatively complex (especially when compared to conditional models) and there are no examples in the papers found of Markov approaches incorporating variations in study centre recruitment.

Five articles used recruitment data from existing trials to illustrate their model or establish the structure and parameters used in their models, whilst three presented analysis based on theoretical data only (Table 3). Only two papers tested the fit of their proposed models using trial recruitment data, Anisomov and Williford [7,8]. Wiliford compared the fit of the poisson to the negative-binomial (poisson-gamma) model, while Anisomov focused solely on the poisson-gamma model. They concluded there is some evidence that the poisson-gamma model provides an acceptable fit for trials with more than 200 participants 8] and more than 20 centres [6]. However, Williford questioned its usefulness for any pre-study prediction unless the distribution of changes in patient intake rates can be estimated and modelled.

**Table 3:** Detail of Trial Data Reported in Modelling Papers

Author	Used Real Life Data?	Where From/How	Was Prediction Useful/authors conclusions in relation to the model and data used
Carter 2004	No	Theoretical example only	Not applicable

Carter 2005	Yes	They used information from a multicentre RCT protocol under development to test and illustrate the three models	The authors conclude that the unconditional approach can yield results not consistent with trial assumptions and may endanger the successful completion of the trial. The models are sensitive to the estimated accrual rate and the conditional and poisson accrual estimation methods maybe useful to researchers designing a complex, multi-center RCT.

Anisimov	Yes	Checked how a poisson-gamma model fits real recruitment data by analysing several tens of completed GSK trials in different therapeutic areas.	Authors conclude that for a sufficiently large number of centres (N > 20) the poisson-gamma model is in good agreement with real data and can serve as a basic recruitment model.

Moussa	No	Theoretical analysis only	Not applicable

Williford	Yes	Analysed weekly patient intake data from 9 multihospital clinical trials coordinated by the Veterans Administration Coordinating Centre between 1975 and 1982. Used the data to review how mean patient intake varies over time within a trial, and whether the assumption of equal patient intake rates throughout a trial is appropriate. Used trial data to compare fit of poisson model to a negative-binomial model	Authors conclude: 1. poisson distribution did not always provide an acceptable fit, nor was the assumption of equal intake rates over three time periods in a trial acceptable. 2. There is some evidence that the negative binomial distribution might provide an acceptable fit for trials with more than 200 patients. However, this distribution does not lend itself to any pre-study prediction unless the distribution of changes in patient intake rates can be estimated and modelled

Gajewski	Yes	To illustrate the proposed model the authors use data from the Kansas University DHA outcome study which is a single centre trial. They take enrolment dates of the first 41 patients recruited and use these to predict the time needed to recruit the remaining 309 patients.	Unclear, the authors don't discuss how long the trial actually took to recruit all patients and compare that to the time predicted from the model. They use the data to illustrate the model only.

Abbas	No	Hypothetical examples presented	Not applicable

Haidich	Yes	A retrospective analysis of database of all 782 clinical studies launched by the AIDS Clinical Trials Group between Oct 1986 and Nov 1999 to identify factors which affect recruitment (use simple regression and multivariate first-order autoregressive model). Model not applied to other trials.	Authors conclude modelling enrolment rates may be used to comprehend long-term patterns and to perform future strategic planning.

## Discussion

There are two significant drivers to overall recruitment in pragmatic multicentre randomised controlled trials unable to take advantage of a pre-existing network of recruiting centres: recruitment of patients, and recruitment of centres.

All of the studies identified primarily considered recruitment of patients. Five of the 8 studies did not consider the role of centre recruitment at all. The others discuss this as an issue, but make no explicit effort to include this important parameter in their model.

The HTA programme experience has been that poor centre recruitment (often due more to issues of process than clinician engagement) is a significant issue in causing trials to miss their recruitment targets.

For example, SANAD - a trial comparing various anti-epileptic agents - ran into difficulties in expanding its centres due to competition from other trials in epileptics underway at the same time [12]. The trial managed to attain 90 centres, but not as quickly as was originally anticipated.

PAC-MAN, an investigation into the use of pulmonary artery catheters in intensive care units had difficulty recruiting centres due to the procedure which was then in place for seeking ethics approval [13]. Of 95 intensive care units which expressed an interest in participation, only 79 eventually completed all the required processes to join the trial, and only 65 recruited at least one patient. At one stage the trial dropped to only recruiting 40% of the predicted number of patients.

REFLUX was a trial investigating surgery in gastro-oesophageal reflux disease [14]. Similarly to PAC-MAC [13] difficulties with ethics resulted in the slow accumulation of centres, resulting in an extension of both time and money in order for the trial to meet its patient recruitment targets.

This review is not without limitation, for example, literature searches may not have identified unpublished or grey literature. While we are confident that we have complete coverage of relevant studies in the formal indexes, it would not be surprising had we failed to identify relevant information in the grey literature - especially anything which was not published in English. However, it seems to us likely that we have identified at least one example of each of the classes of approach which could be used to address this issue.

A further limitation may be the assessment criteria against which we judged the models. These were derived from discussions within the scientific secretariat at the NIHR Evaluation, Trials and Studies Coordinating Centre. This is the group which supports the HTA programme - consequently it is possible that the criteria that we used give emphasis to aspects of models which the HTA programme would particularly value. Had these criteria been developed by another group they may have developed a differing set.

In order for a model to be useful, trialists need to take into account local factors which influence a model's parameters. Examples would include delays in trialist and centre start times caused by variations in local ethics and local governance approvals or the existence of trials competing for the same patient pool. Delays caused by governance processes could be estimated from the previous experience of trialists or programmes making decisions. Patient competition must be estimated from knowledge of ongoing or planned trials, potentially run or funded by other groups and estimates of those factors could be used to adjust the parameters used in the predictive model - for example by reducing the expected number of patients recruited while a competitive trial is underway.

It is in the nature of pragmatic trials that an ad-hoc set of centres is required for each trial. The topics commissioned by the programme are so varied that a standing trial network would not usually be useful. There are advantages to be gained from a standing network - experience, habituation to trial recruitment and a pool of identified potential trial participants being examples. It is hoped that the NIHR research networks will in future aid with recruitment to trials in the UK- fulfilling in part the role of a standing trial network.

There appears to be a gap for a tool to predict recruitment to clinical trials of ad-hoc networks, which would account for both centre and patient recruitment, and recognise that one will drive the other. Such a tool might be built on the conditional, Poisson or Poisson-gamma models - although the model comparison in Table 3 suggest that the conditional model is the leading candidate. Bayesian models, however, show promise for use during the conduct of a trial.

The possible attraction of a more sophisticated model is the confidence interval it can place around the accrual period estimate. The major trade-off would be the increased complexity of use of the Bayesian or Poisson based models compared to the relative simplicity of the deterministic conditional model. Given Carter's (2005) findings that there was very little difference in prediction between the Poisson and conditional models he used, the relative simplicity of the conditional model may be more attractive [3]. It should be noted that all the models are heavily dependent on the parameters used (e.g. estimates of monthly recruitment of participants). The confidence intervals from the Bayesian and Poisson models do not reflect the inaccuracy of the model parameters - but more the distributions described by those parameters. The provided confidence levels may therefore offer a false degree of security in the confidence of the estimates produced by the models.

The ease of implementation of a model would also contribute to which method should be used. A model which could be operated by an applicant on their own computer, or alternatively online, might be preferred over one which required significant statistical expertise to implement and use. Alternatively, recruitment prediction could become a service offered by local Clinical Trials Units, or Research Design Services, which may allow more sophisticated tailoring of models to the particular circumstance which a trialist encounters.

The NIHR research programmes do not currently have a preferred model for clinical trial prediction, consequently most applicants choose the simplest - the unconditional model. The programmes could give consideration to giving guidance on prediction of recruitment to applicants - and especially for them to include centre recruitment in their model. They may wish to suggest appropriate methods to use for recruitment prediction, mandate a particular existing model, or design their own model. As we move towards developing a new model, we will have to consider the conflicting needs for simplicity and estimation - possibly by developing more than one model and testing which gives the more useful predictions. Any model chosen should be validated against both retrospective and prospective data, to ensure the predictions it gives are superior to those currently used.

## Conclusion

To meet the needs of a number of research programmes, a new model is required as a matter of importance. Any model chosen should be validated against both retrospective and prospective data, to ensure the predictions it gives are superior to those currently used

## Abbreviations

CTU: Clinical Trials Unit; HTA: Health Technology Assessment; ICU: Intensive Care Unit; NIHR: National Institute for Health Research; PAC-MAN: Pulmonary Artery Catheters in patient MANagement; RCT: Randomised Controlled Trial; RDS: Research Design Service; REFLUX: The effectiveness and cost-effectiveness of minimal access surgery amongst people with gastro-oesophageal reflux disease - a UK collaborative study; SANAD: StANdard versus new Antiepileptic Drugs.

## Competing interests

All three authors are employed by the NIHR Evaluation, Trials and Studies coordinating centre, who manage the NIHR HTA programme.

## Authors' contributions

KB designed and led the literature review, and wrote the first draft of the paper.

LD suggested the categories for the review, reviewed the papers and contributed statistical advice and contributed to the writing of the paper.

AC conceived the need for a recruitment prediction model for the NIHR HTA programme, contributed advice, and reviewed papers for the literature review.

All authors read and approved the final manuscript.

## Pre-publication history

The pre-publication history for this paper can be accessed here:

http://www.biomedcentral.com/1471-2288/10/63/prepub

## Supplementary Material

Additional file 1 **Summary of Study Data Extraction**. summary of data extraction from selected papers. Click here for file
