# Supplementary material for: A systematic review of models to predict recruitment to multicentre clinical trials
Source: BMC Med Res Methodol. 2010 Jul 6;10:63. doi: 10.1186/1471-2288-10-63 (PMC2908107; doi:10.1186/1471-2288-10-63)
Supplement: Additional file 1 — Summary of Study Data Extraction. summary of data extraction from selected papers. [file 1471-2288-10-63-S1.DOC]

Summary of study data extraction

| **Paper** | **Question Addressed** | **Applicability** | **Approach** | **Centre Recruitment** |
| --- | --- | --- | --- | --- |
| Carter (2004) (4) | What is the optimum duration for the recruitment phase of a clinical trial? | Allows for centre recruitment over time, and ad-hoc networks of recruiting centres. | Data is simulated from a Poisson distribution and used to estimate the time needed to recruit a predetermined no. of patients, with a certain level of confidence (e.g. 90% certain will recruit the required no. of patients within 580 days).  The paper does not report a real life use. | Allows for centre recruitment over time, but gives no suggestions on how to predict centre recruitment – this must be given when the model is set up. |
| Carter (2005) (3) | How can the required accrual period be estimated when a trial is being planned? | Allows for centre recruitment over time, and ad-hoc networks of recruiting centres. | Discusses three approaches:  The unconditional approach assumes each planned centre will start recruiting on day one at a constant rate.  The conditional approach takes into account likely starting dates for centres.  The Poisson process is that discussed in Carter’s previous paper (1), and two different methods of use are discussed.  The three approaches are compared, but the predictions are not validated against what actually happened. | Allows for centre recruitment over time, but gives no suggestions on how to predict centre recruitment – this must be decided when the model is set up. |
| Anisimov (2007) (7) | 1) How can the required accrual period best be estimated when a trial is being planned?  2) For ongoing trials can we predict the remaining recruitment time using current recruitment information?  3) How many additional centres should be added to ensure there is a high probability that the no. of participants will be recruited by a fixed date? | Applicable to a pre-established network of centres. Makes no allowances for centres joining or leaving the recruitment pool. | Propose a recruitment model where the patients arrive at different centres according to Poisson processes, with the rates viewed as a sample from a gamma distribution. Assumes a predetermined number of recruiting centres except when answering question 3. Generates estimates with confidence intervals | No – the models developed assume for simplicity that all centres will be initiated on the same day |
| Moussa (1984) (6) | How can a trial best be planned within the constraints of available time and available resources (both financial and patient)? | Takes a wide view of clinical trial planning. Starts from the very basics of sample size calculation, and discusses how to base a sample size on multiple goals – including cost.  Discusses recruitment prediction for both fixed and variable rates, but doesn’t relate these rates to centre recruitment. | Takes a deterministic approach to calculating recruitment in a given period of time, uses normal distribution Z values. | Doesn’t explicitly consider centre recruitment, but the variable rate recruitment prediction could be adapted to explicitly consider centres. |
| Williford (1987) (8) | How can future trial recruitment best be predicted, given past recruitment in the same trial? | Attempts to learn from past experience to inform future predictions. | Compares a Poisson distribution, negative binomial distribution and Baysian approach to prediction of future recruitment. | Does not consider centre recruitment. |
| Gajewski (2007) (9) | Given information on accrual at a particular point in a trial, how can we predict future accrual? | Is perhaps more useful for monitoring a trial once it has started. Does not consider ad-hoc centre networks. | Uses Bayesian methods to determine the average waiting time between participants, the prior distribution assumed is the inverse gamma and the likelihood distribution assumed for waiting times is the exponential distribution. Relies on either prior experience or investigator best guess for the initial estimates, which are then refined as data becomes available.  The paper discusses applicability in a real life example -the KUDOS study. (16) | The possibility of new centres is discussed, but not explored. The possibility that centres may not all start immediately is not considered. |
| Abbas (2007) (10) | How can we recruit the maximum number of patients in the minimum amount of time? | Doesn’t consider the limitation of resources inherent in most public sector trials – assumes accrual rate is all important.  Derives expected accrual rate from previous recruitment experience – which may not be practical with ad-hoc centre recruitment. | A Markov model and Monte Carlo simulation, using both continuous and discrete time assumptions.  No use of the model in a real life situation is reported. | There is no consideration of centre recruitment. |
| Hadich (2001) (11) | Can quarterly patient enrolment be modelled in terms of predictors? | Used a dataset from an established network of HIV trial centres.  Addresses seasonal and other variation in recruitment rates, rather than the overall recruitment profile | Time series modelling (first order autoregressive model) of 872 trials launched by the AIDS clinical trials group to assess what factors affect recruitment to trials. Factors considered include time of year, season, launch of new studies, number of studies starting each quarter and early starting effect. No prospective validation. | Discusses that centres may not start at the same time so assess in their model whether an indicator variable for the first 3 quarters of recruitment helps predict recruitment (it doesn’t). No consideration of centre recruitment. |
